# Pan-India novel coronavirus SARS-CoV-2 genomics and global diversity analysis in spike protein

**DOI:** 10.1016/j.heliyon.2021.e06564

**Published:** 2021-03-19

**Authors:** Shweta Alai, Nidhi Gujar, Manali Joshi, Manish Gautam, Sunil Gairola

**Affiliations:** a Department of Health and Biological Sciences, Symbiosis International University, Pune, Maharashtra, 412115, India; b Bioinformatics Centre, Savitribai Phule Pune University, Pune, Maharashtra, 411007, India; c Serum Institute of India Pvt Ltd, Pune, Maharashtra, 411028, India

**Keywords:** Receptor binding domain, COVID-19, SARS-CoV-2, Pandemic, Comparative genomics, Fatality rate, Clades, Neutralizing antibodies

## Abstract

The mortality rates due to COVID-19 have been found disproportionate globally and are currently being researched. India mortality rate with a population of 1.3 billion people is relatively lowest to other countries with high infection rates. Genetic composition of circulating isolates continues to be a key determinant of virulence and pathogenesis. This study aimed to analyse the extent of divergence between genomes of Indian isolates (n = 2525 as compared to reference Wuhan-1 strain and isolates from countries showing higher fatality rates including France, Italy, Belgium, and the USA. The study also analyses the impact of key mutations on interactions with angiotensin converting enzyme 2 (ACE2) and panel of neutralizing monoclonal antibodies. Using 1,44,605 spike protein sequences, global prevalence of mutations in spike protein was observed. The study suggests that SARS-CoV-2 genomes from India share consensus with global trends with respect to D614G as most prevalent mutational event (81.66% among 2525 Indian isolates). Indian isolates did not reported prevalence of N439K mutation in receptor binding motif (RBM) as compared to global isolates (0.54%). Computational docking and molecular dynamics simulation analysis of N439K mutation with respect to ACE 2 binding and reactivity with RBM targeted antibodies viz., B38, BD23, CB6, P2B–F26 and EY6A suggests that variant have relatively higher affinity with ACE 2 receptor which may support higher infectivity. The study warrants large scale monitoring of Indian isolates as SARS-CoV-2 virus is expected to evolve and mutations may appear in unpredictable way.

## 1 Introduction

The current 2019 coronavirus pandemic (COVID-19) is caused by a positive RNA virus, referred to as severe acute respiratory syndrome coronavirus 2 (SARS-CoV-2) [1]. Despite all the global efforts, the virus continues to spread and infect a large population and has affected 218 countries, with more than 101,053,721 confirmed cases, 2,182,867 deaths globally (WHO, 29 Jan 2021) [2]. One of the most striking features of COVID-19 is the variable mortality rate across countries [3]. Global case fatality rates (CFR) varied from 0.06 to 18.94%. France recorded the highest CFR of 18.65%. Belgium and Italy had double-digit CFR rates, while the United States, which experienced the largest outbreak, had a CFR of 5.76%. India, the second most populated country, reported 7,761,312 cases in all with 117,306 deaths (WHO, October 23) [4]. Trends in mortality rates in India are intriguing, despite the second largest reported cases of infection ("https://covid19.who.int/table"), second after the USA (more than 76 lakh) in terms of total confirmed cases. Although numerous hypotheses are proposed, one hypothesis includes the variability of circulating viral strains. Monitoring this variability in key viral genomes and immune-dominant antigens may explain the infectivity, pathogenesis, and transmission of SARS-CoV-2 [10].

Genome structure of SARS-CoV-2 is characterized by a size of around 30 kB with a large gene region (ORF) encoding non-structural proteins (NSP) and the genes encoding spike (S) glycoprotein, envelope protein, membrane (M) glycoprotein and nucleocapsid (N) proteins [7]. SARS-CoV-2 transient glycoprotein contains a region, the receptor binding domain (RBD), which specifically identifies the human angiotensin conversion enzyme (ACE2) as its receptor [8]. Antibodies targeting spike proteins, especially against the RBD are neutralizing in function [9]. Mutations that lead to variations in these hot spots may affect infectivity, pathogenesis, and transmission of SARS-CoV-2 [10,72,79].

Rapid sequencing of the SARS-CoV-2 genome globally has greatly facilitated efforts to understand global epidemiology [5, 71]. Since the first SARS-CoV-2 reference genomic sequence was reported in January 2020, more than 1,58, 776 global sequences have been deposited into GISAID (23/10/2020, https://www.gisaid.org).

We report here comparative genomics of Pan India isolates (n = 2525) vis a vis reference genome and isolates from countries showing high fatality rates including France, Italy, Belgium, and the USA. The study also reports deep scanning of mutational events and their global frequency at each amino acid residue in spike protein using 1,44,605 global sequences submitted at GISAID. In-silco studies on impact of mutations on structure and binding affinity to critical human ACE2 receptor [73] and RBM targeted antibodies B38, BD23, CB6, P2B–F26 and EY6A were also carried out using molecular docking and simulation analysis [11].

## 2 Materials and methods

### 2.1 Genome sequences retrieval and alignment

A total of 2525, SARS-CoV-2 complete, and high coverage viral genome sequences were downloaded from Global Initiative on Sharing All Influenza Database (GISAID) platform (23/10/2020). A total of 11,302 genome sequences from countries representing France, Belgium, Italy, India, and the USA (North America) were downloaded from GISAID (https://www.gisaid.org/) (collected till 6/0/2020) and compared for dominant clade analysis [16]. Viral genomes with human hosts were selected, excluding low coverage and incomplete (<29,000 nucleotides) genomes. For phylogenetic analysis 863 complete and high coverage sequences from India were used and compared with sequence from GISAID as hCoV-19/Wuhan/WIV04/2019/EPI_ISL_402124 reference genome. The reported and novel mutations were catalogued. (Additional File1). Lineages were predicted using Pangolin COVID-19 lineage assigner (https://pangolin.cog-uk.io/) [17]. The 1,44,605 spike protein sequences were retrieved from GISAID and compared with reference spike protein sequence (SARS-CoV-2 spike glycoprotein (EPI_ISL_402124).

### 2.2 Multiple sequence alignment and phylogenetic analysis of Indian isolates

Sequences from GISAID were downloaded and consensus sequences were aligned using MAFFT [18]. A maximum-likelihood phylogenetic tree was constructed using IQ-TREE and visualized with iTOL [19, 20].

### 2.3 Structure prediction and docking analysis

Impact of mutations on binding affinity towards human ACE2 receptor was performed using docking and MD simulations analysis. The structures of wild type SARS-CoV-2 Spike RBD domain complexed with host ACE2 receptor was retrieved from Protein Data Bank (PDB ID: 6LZG) [21]. Spike protein was mutated using UCSF Chimera 1.10 software [22]. Mutant structures were retrieved from Chimera and energy was minimized for further analysis. All the wild and mutant structures were docked using ZDOCK docking server [23]. Docked structures were subjected to energy minimization and the binding energy was calculated using PDBePISA [24]. The structures of wild type SARS-CoV-2 Spike RBD domain complexed with antibodies B38,BD23,CB6, P2B–2F6, EY6A was retrieved from Protein Data Bank (PDB ID: 7BZ5, 7BYR, 7CO1, 7BWJ and 6ZER) respectively [12, 13, 14, 25].

### 2.4 Modelling of the wild type and N439K variant with respect to ACE-2 binding

The structures of wild type SARS-CoV-2 Spike RBD domain complexed with host ACE2 receptor was retrieved from the Protein Data Bank (PDB ID: 6LZG) [21]. Spike protein was mutated using UCSF Chimera 1.10 software [22]. These structural models were used for molecular dynamics simulations. All molecular dynamics (MD) simulations were performed with GROMACS 2019 [74] software using CHARMM36 forcefield [75]. Explicit TIP3P water model was used to represent the water molecules. To neutralize the systems Na^+^ and Cl^−^ ions were added. Energy minimization was performed using the Steepest Descent algorithm for 50000 steps. The system was equilibrated under the NVT conditions for 100 ps followed by NPT equilibration of 1000 ps. Temperature coupling was applied to maintain the system temperature at 300 K using the velocity rescaling algorithm. Semi-isotropic pressure was maintained using Parrinello Rahman pressure coupling with a pressure of 1 bar. Atomistic simulations were run for 100 ns for both the systems. Trajectories were viewed and analysed using Visual Molecular Dynamics (VMD) [75] tool. Standard GROMACS tools were used to plot the Root Mean Square Deviation (RMSD) and Root Mean Square Fluctuation (RMSF) of the system [77]. Hydrogen bond analysis was performed in VMD using Hydrogen bonds plugin. Binding free energies were calculated using MM-PBSA program in GROMACS [76].

## 3 Results

### 3.1 Genomic characterization of Indian SARS-CoV-2 genomes centric to global population structure

#### 3.1.1 Diversity within Indian SARS-CoV-2 genomes compared with reference strain

To understand the diversity in the SARS-CoV-2 isolates from India as compared to reference strain (hCoV-19/Wuhan/WIV04/2019/EPI_ISL_402124), we analysed a total of 2525 SARS-CoV-2 genomes retrieved from global dataset GISAID (23/10/2020) [26]. The samples were isolated between February–October 2020. A total of 2525 complete or near complete genome sequences with high coverage deposited from India were used in this study. The complete characteristics of 2525 SARS-CoV-2 genomes sequences are provided in additional file 1.

**Figure 1:**
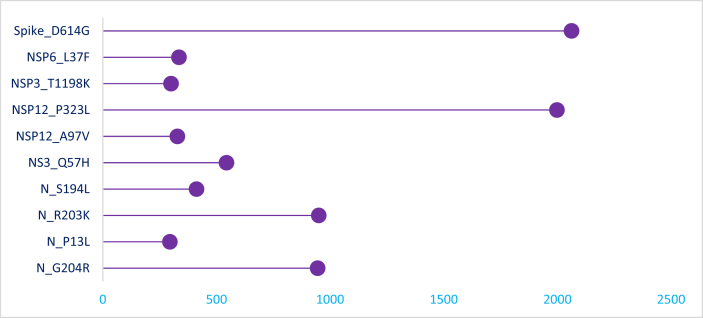
Lollipop plots showing mutations distribution and frequency in Indian SARS-CoV-2 genome sequences. The frequency of mutations is shown on the X-axis and the presence of a mutation is shown on the Y axis (lollipop), correlates with the heights of the vertical lines representing each lollipop.

The prevalent markers of SARS-CoV-2 diversity observed in Indian genomes are shown in Figure 1. A total of 954 variants in Indian genomes were observed, 659 were observed as a single event. A23403G and C14408T were observed at higher frequencies (>50%) in all the genomes, while G25563T, C13730T, G11083T C6312A, C241T, C3037T, G11083T, C13730T, C28311T, C6312A and C23929T mutations were predominated (>24% frequency) in Indian genomes (Additional File1).

**Figure 2:**
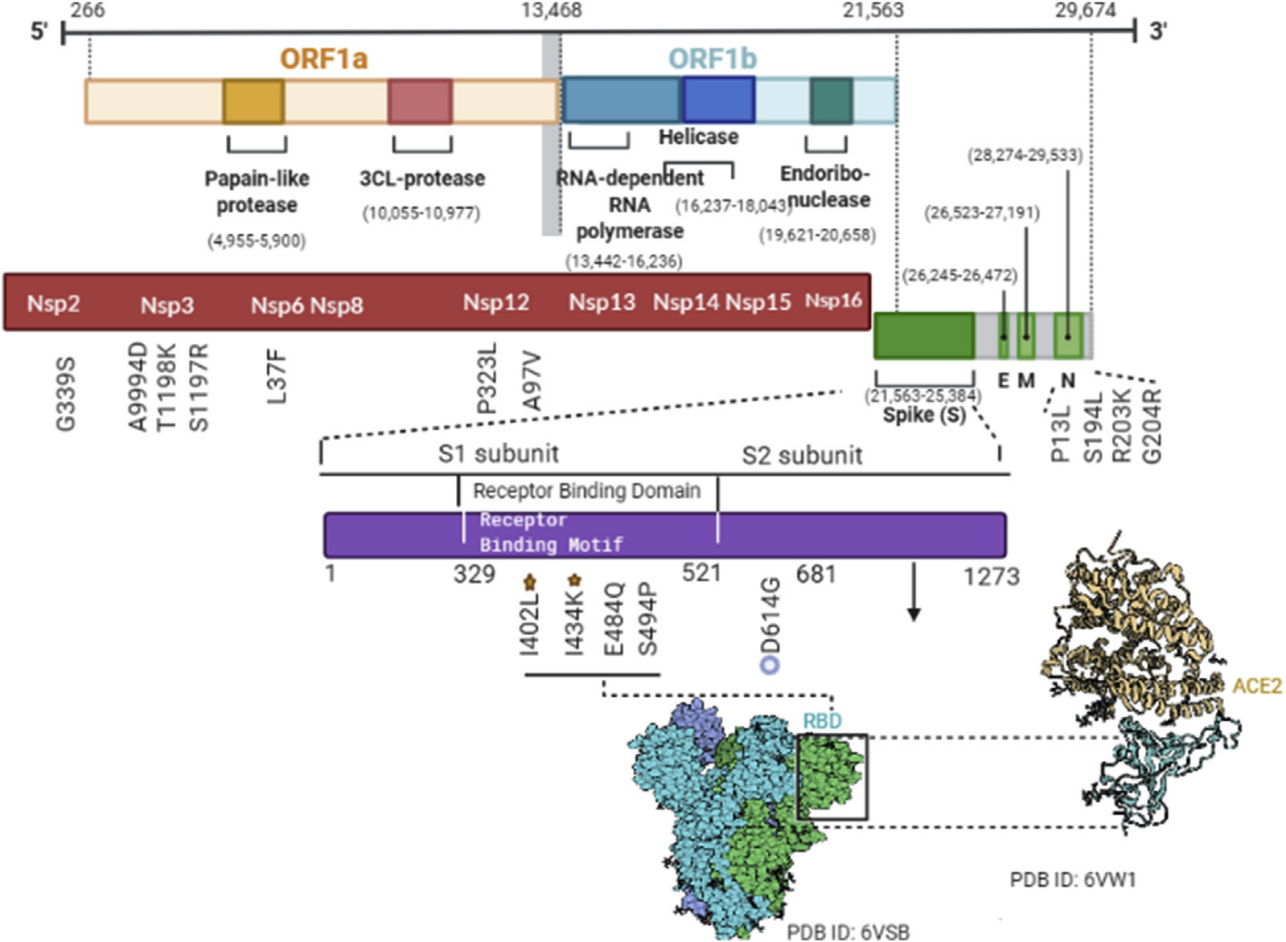
The linear diagrams represent mutations observed in genome and its genes distribution in the SARS-CoV-2 Indian genome sequences. Diagrams in red and violet represent the protein subunits of ORF1ab and S, respectively. The presence of a mutation is represented in front of gene under each line, the most frequent variants in RBD domain are annotated as star mark the amino acid change at that specific site, and most frequent of Spike protein and among all mutations is presented by circles.

Higher frequency mutations were mapped for key SARS-CoV-2 protein structures, including nucleocapsid, nsp3, nsp12, nsp14, nsp2 and spike protein (Figure 2). Out of 954 events, 585 events were found in open reading frame1ab (ORF1ab), which is the longest ORF occupying two thirds of the entire genome. ORF1ab is transcribed into a multiprotein and subsequently cleaved into 16 non-structural proteins (NSPs). Of these proteins, NSP12 has the largest number of variants including, P323L as dominant (n = 1998) followed by A97V (n = 328). D614G mutation in spike protein, which is considered as a prevalent global mutation, was present in 2062 of the 2525 (81.66%) sequenced genomes [27, 28].

#### 3.1.2 SARS-CoV-2 genomes diversity within India

**Figure 3:**
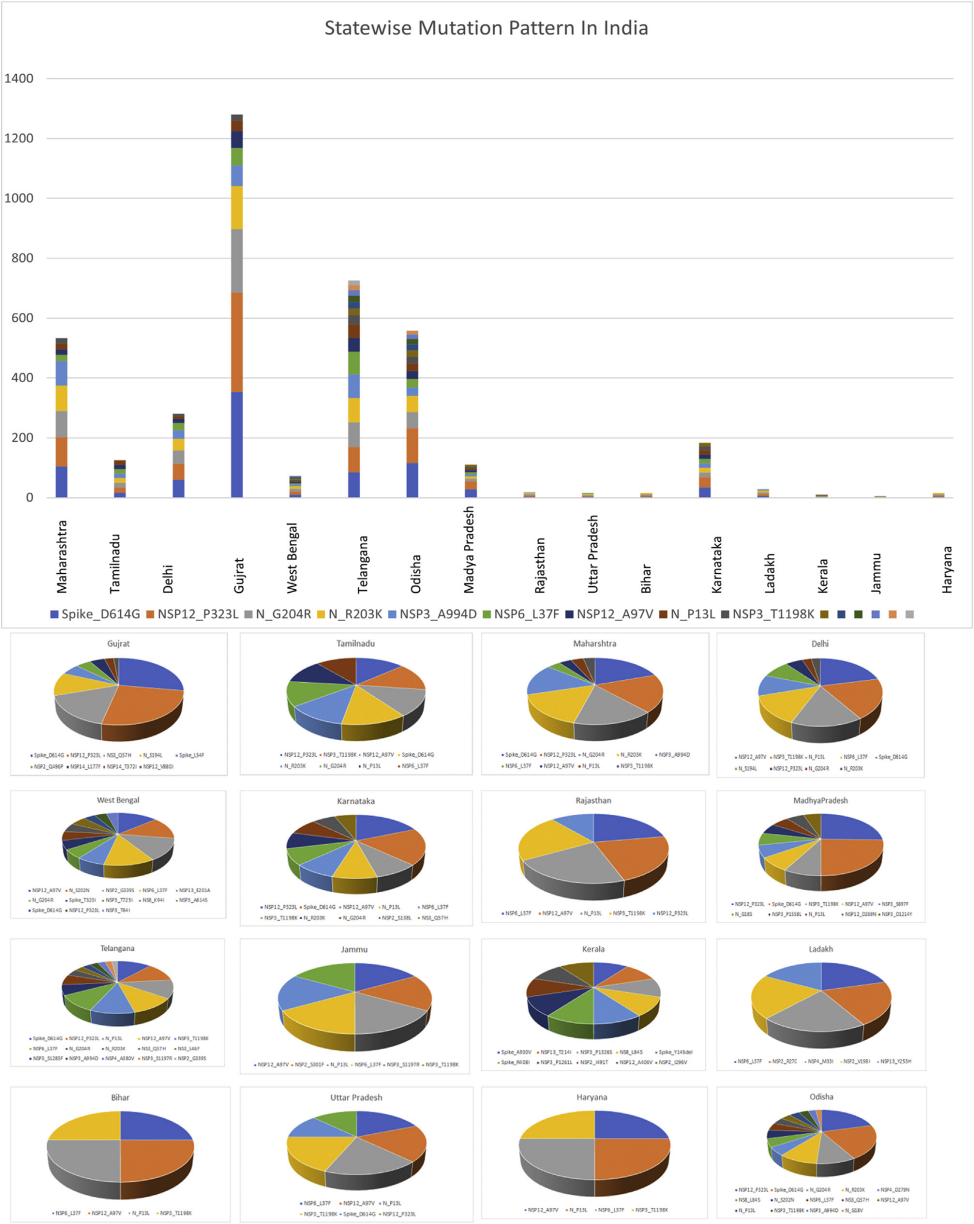
Statewise mutation prevalance of SARS-CoV-2 genomes in India. A) Graph showing combine statewise mutation marker prevalence of SARS-CoV-2 sequences collected from states within India. B) Pie chart showing mutation marker prevalence in each individual states of SARS-CoV-2 sequences collected from states within India.

The SARS-CoV-2 collection from India submitted at GISAID consisted of ~2525 isolates after excluding low coverage and incomplete genomes sequences (<29,600 bases). The isolates belonged to 17 different states: 369 (30.75%) isolates from Gujrat (GJ), 169 (14.08%) isolates from Telangana (TS), Odisha 166 (13.83%) and 140 (11.66%) isolates from Maharashtra (MH) (Additional File 1). The mutational patterns prevalent in each state are presented in Figure 3. Maharashtra recorded highest number of cases (n = 2, 92,589) followed by Tamil Nadu (n = 16, 0907) and Delhi (n = 12,0107) as of 17 July 2020. The dominant mutation observed in Maharashtra, Tamil Nadu and Delhi was D614G, NSP12 (P323L) and NSP12 (A97V) respectively. Prevalent mutation observed in Maharashtra was in spike protein D614G (75%), followed by non-structural protein NSP12 (P323L) and in nucleoprotein (G204R, R203K). Prevalence of D614G observed in Tamil Nadu and Delhi was less as compared to Maharashtra. The statewise list of mutations observed in country is provided in additional file 2.

Regardless of fourth highest in the number of reported cases, Gujrat has highest death rate in the country as 6.2% which is double than the global mortality rate evaluated by WHO which 3.2% [2] was. Gujrat followed by Madhya Pradesh and West Bengal both of which have an estimated death rate of 4.2%. Gujrat and Madhya Pradesh showed similar trends in prevalent mutations in spike (D614G) and NSP12 (P323L), where West Bengal showed NSP12 (A97V), N (S202N), NSP2(G339S) mutations predominately (Figure 3). Statewise prevalence of marker variant differed significantly (Figure 3). Statewise mutation analysis suggests predominance of mutation in spike D614G with increased transmission and infectivity.

#### 3.1.3 SARS-CoV-2 genomes diversity in India as compared to global population

To understand the global positioning, we compared SARS-CoV-2 genomes from India along with the 50,500 global sequences from GISAID [26]. Global characterization of the SARS-CoV-2 variants from all ~50,500 viral genomes sequences, suggested three major variants groups as 1,771 isolate Group 1 “C241T, C3037T, C14408T, A23403G, G28881A, G28882A, G28883C”, the 1,458-isolate Group 2 “C241T, C1059T, C3037T, C14408T, A23403G, G25563T”, and the 727-isolate Group 3 “C241T, C3037T, -C14408T, A23403G”. The four most common mutations were (C241T/5UTR in orf1ab, C3037T in orf1ab (F924F, C14408T P4715L), and D614G in spike protein [29]. Mutations C241T, C3037T, A23403G and C14408T were observed at higher frequencies (>50%) in Indian genomes consistent with global trends. However, co-evolving mutations observed in developed countries associated with clades G,GH and GR such as G25563T (ORF3a), C26735T (NSP14) and C18877T (M protein) were observed less frequently (<15%) in Indian genomes. The most common variant observed globally, 3037C > T, ORF1ab: P4715L, RdRp: P323L; and D614G mostly reported from Europe and the USA were also observed in Indian population [30]. Other key variants including ORF3a: Q57H, ORF1ab: T265I (NSP3: T85I), ORF8: L84S, N203 (204del-insKR), ORF1ab: L3606F (NSP6, L37F) were also observed in genomes retrieved from India [31].

### 3.2 Phylogeny and lineage analysis

To study, SARS-CoV-2 classification for their multitude of distribution across geographical locations of India, vis-à-vis states of India, we explored the lineages spread across country. We further observed the prevalence of different clades within high fatality rate populations.

#### 3.2.1 Viral clade analysis

**Figure 4:**
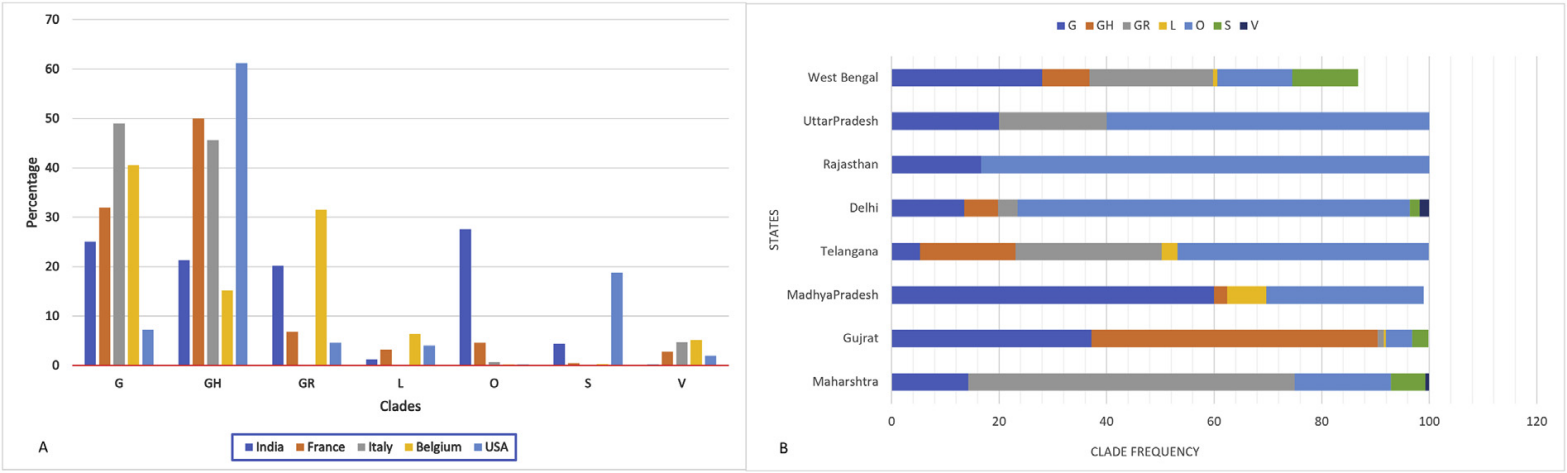
Regional Clade distribution of SARS-CoV-2 genomes. A) Graph showing country wise clade distribution of sequences collected from countries Belgium, Italy, France, India, and the USA. The charts show the clades grouped on X axis and countries represented in different colours with relative frequencies of clades for each country on Y axis. The countries are colour coded in squares and shown below chart in the diagram. B) Graph showing statewise clade distribution of sequences collected from India. The charts show the relative frequencies of clades for each state on X axis and states with different clades represented on Y axis.

The global isolates were assigned to major GISAID clades according to the marker variant and existing GISAID clades designations [32, 33, 34]. These clades were characterized in the context of marker variant relative to the reference strain (hCoV-19/Wuhan/WIV04/2019/EPI_ISL_402124) namely, clade S, L, V, G, GH, GR, and other clades. Clades are characterized as S (C8782T, T28144C, NS8-L84S), L (C241, C3037, A23403, C8782, G11083, G25563, G26144, T28144, G228882), V (NSP6-L37F, NS3-G251V), G (S-D614S), GH (S-D614S, NS3-Q57H), S (S-D614S, NG204R). The most represented clades were clade GR observed in 11,298 complete genomes (GISAID, 18/06/2020) [35]. The clade was more prevalent in genomes sequenced from South America and Europe. The second most frequent clade represented was GH which was observed frequently from the genome sequences in North America and Africa continents [36]. The most represented clade from the Asia region was categorized as ancestral type O, which is similar with originating strain from Wuhan, China [6]. Examining the variants from the O clade isolates observed the most frequent variant as G11083T (46.7%), C28311T (22.7%), and C13730T (20.4%). Clades analysis of the SARS-CoV-2 genomes from India classified under 7 clusters a identified by GISAID and Nextstrain consortium as: G, GH, GR, S, L, V and O. The first and the major cluster encompassed 320 (27%) of genomes which fell into the O clade of the SARS-CoV-2 genome (Additional file 3). The clade distribution was represented in different states (n = 17) across the country such as Haryana, West Bengal, Maharashtra, Tamil Nadu, Delhi, and Telangana and other states (Figure 4).

**Figure 5:**
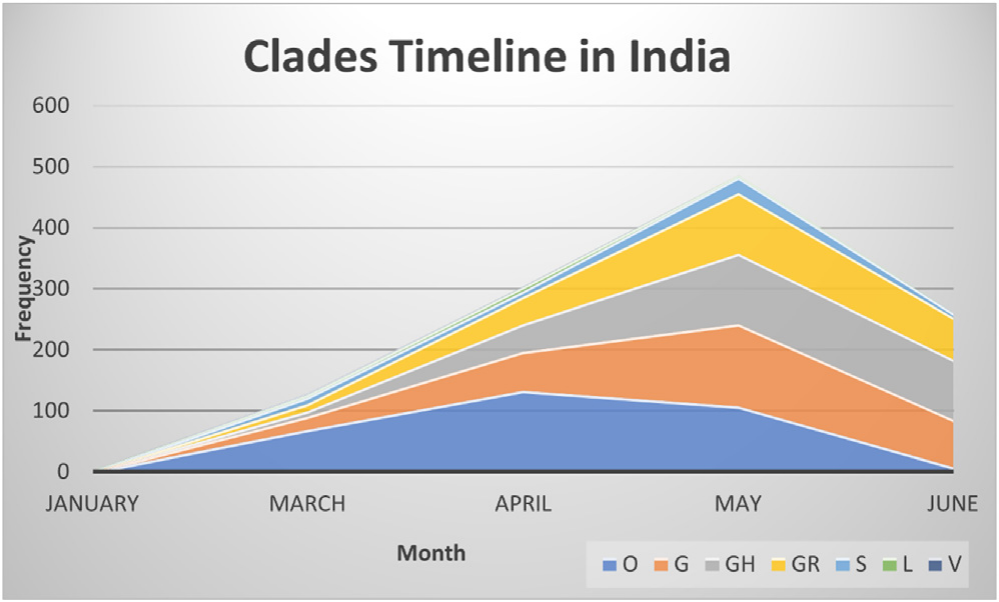
Clade distribution timeline in India. The graph represents distribution of clades in India from January till July 2020.Clades was represented in square box with respective colours below the x axis.

The clades are colour coded in squares and shown above chart in the diagram. As transmission across country is increasing trends in clade predominance was observed during these six months of the pandemic in the country. Ever since the emergence of outbreaks clade “O” is still prevalent, but the clades G, GH and GR were rapidly increasing since ease in social restrictions such as lockdowns and migration of workers across country. The wild type of clade “O” showed gradual decrease in the number of incidences over a time (Figure 5).

Further to study association of fatality rate and predominant clade, we compared a total of 10,188 complete and high coverage genome sequences from countries reported higher fatality rates across the globe such as France, Italy, Belgium, and the USA, showed a dominant clade G in France, Italy, Belgium, and clade GH in the USA (Figure 4) (Additional file 3). Clade G and GH identified by the signature marker D614G variant in spike protein which is increasing its frequency across globe and specially developed countries. Recently reported as per Korber et al, D614G mutation increased infectivity of the virus [27]. India although reportedly third highest country in the world in terms of infected cases of COVID-19, fatality rate is still less as compared to the many developed world (https://www.mohfw.gov.in/). We observed a dominant clade ancestral type “O” in India was dominant during early phase of pandemic. We observed clade G, GH and GR as frequent clade in Gujrat, West Bengal, and Madhya Pradesh respectively (Figure 4). Enrichment and diminution of specific clades was observed in certain states of India [37]. Overall, within genome sequences from high fatality rates of COVID-19 regions, we observed a mutation D614G as prevalent with its resembling clades G, GH and GR.

#### 3.2.2 Phylogenetic analysis

**Figure 6:**
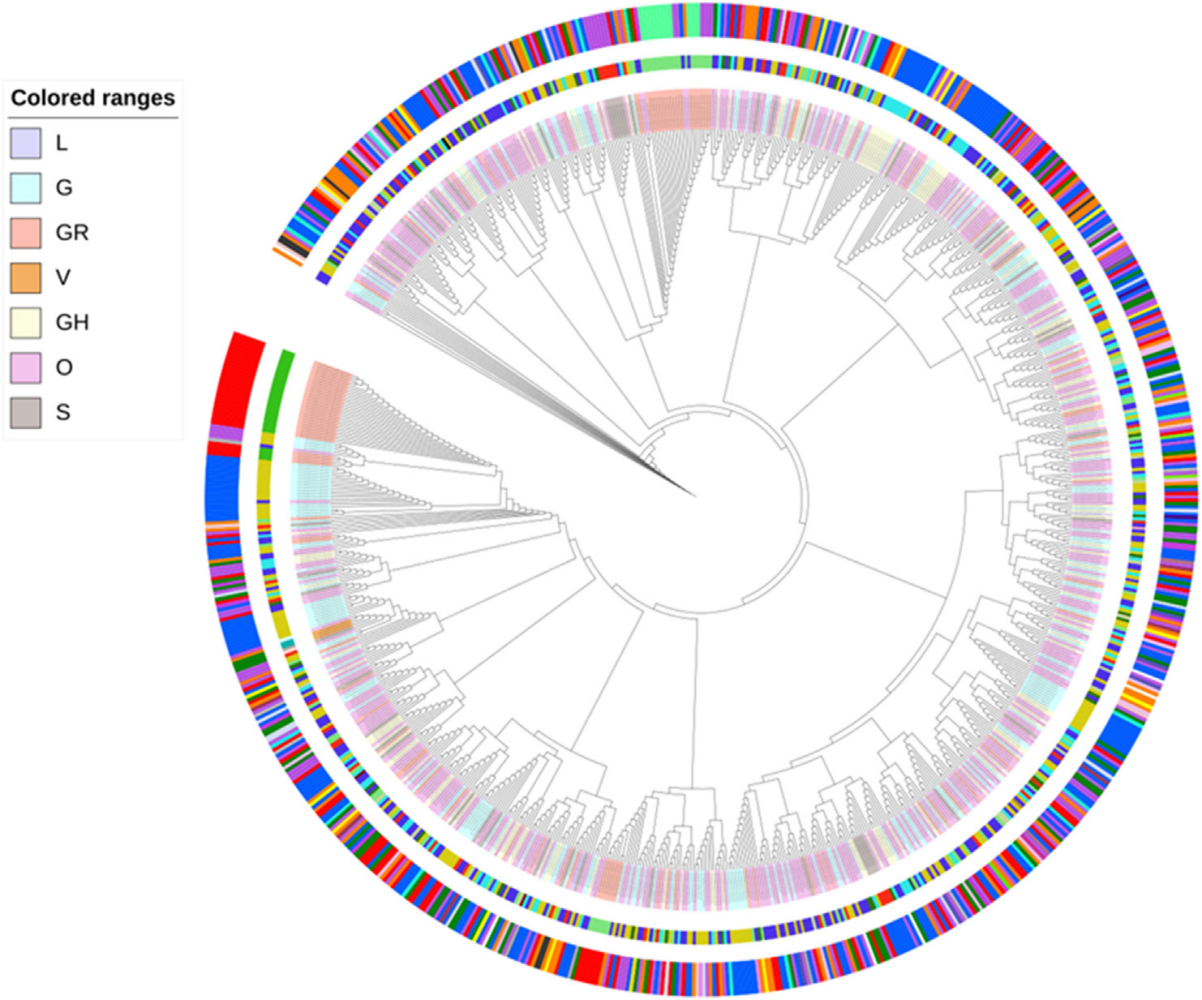
The phylogenetic tree based on the whole genome alignment of 863 genomes sequenced from India. Tree showed the presence of 7 different clades and demonstrates that SARS-CoV-2 is wildly disseminated across 237 distinct geographical location assessed till 19/06/2020. Tree was rooted with hCoV-19/Wuhan/WIV04/2019/EPI_ISL_402124 as a reference. Clades were represented as coloured ranges shown in left panel of the diagram. First outer circle represents lineages corresponding which are represented as strip label. Outer circle represents coloured labels representing different geographical regions from India; Maharashtra-Orange, Gujarat- Blue, Delhi- Green, Karnataka- Yellow, Telangana- Red, West Bengal- Purple, Uttar Pradesh- Green, Haryana-Grey.

Phylogenetic analysis based on whole genome alignment of Indian genomes was performed using maximum likelihood tree rooted against reference hCoV-19/Wuhan/WIV04/2019/EPI_ISL_402124 (Figure 6). Phylogenetic analysis of 864 genomes was done as per the definitions of the PANGOLIN lineage and GISAID clades [32]. The overall 17 different lineages were observed (A, A.1, A.2, A.3, B, B.1, B.1.1, B.1.18, B.1.2, B.1.5, B.136, B.2, B.2.1, B.2.2, B.4 and B.6) of the virus circulating in India. The dominant lineages observed was B.1.36 (n = 184), B.1 (n = 143), A (n = 14), B.6 (n = 12), B.1.1 (n = 5), B (n = 3). Clade distribution highlights the dominant prevalence of clades as GH (n = 187), G (n = 139), O (n = 17), 103 S (n = 13), GR (n = 4) and L (n = 1) in Indian population (file 4). Clades were distributed across different geographical locations within country, which demonstrate that SARS-CoV-2 is wildly disseminated across distinct states in India. Phylogeny indicate occurrence of cluster transfer events across populations. Lineages observed India are clustering with sequences from Asia, Europe, the USA, and other Asian countries indicating multiple introductions of multiple lineages of the virus into the country [36]. (Additional File 4).

### 3.3 Global SARS-CoV-2 spike protein variation analysis

#### 3.3.1 Comprehensive mutational scanning and its global prevalence of spike protein mutations

Spike glycoprotein (S protein) of SARS-CoV-2 mediates receptor recognition and membrane fusion with the host cell [38, 39]. During viral infection, the trimeric S protein is cleaved into S1 and S2 subunits and S1 subunits are released which contains the receptor binding domain (RBD), which directly binds to the peptidase domain (PD) of angiotensin-converting enzyme 2 (ACE2) [40]. Whereas S2 is responsible for membrane fusion. When S1 binds to the host receptor ACE2, another cleavage site on S2 is exposed and is cleaved by host proteases, a process that is critical for viral infection [39, 40, 41]. Many preprint studies reported different mutations at protein level and predicted its possible effect on binding affinity with host receptor [42, 43, 44]. Here, we scanned mutation at each single residue in 1273aa long protein sequence and explored their global prevalence. We also mapped mutation at different sites and regions on spike protein to understand its impact on its role in pathogenicity and disease transmission.

**Figure 7:**
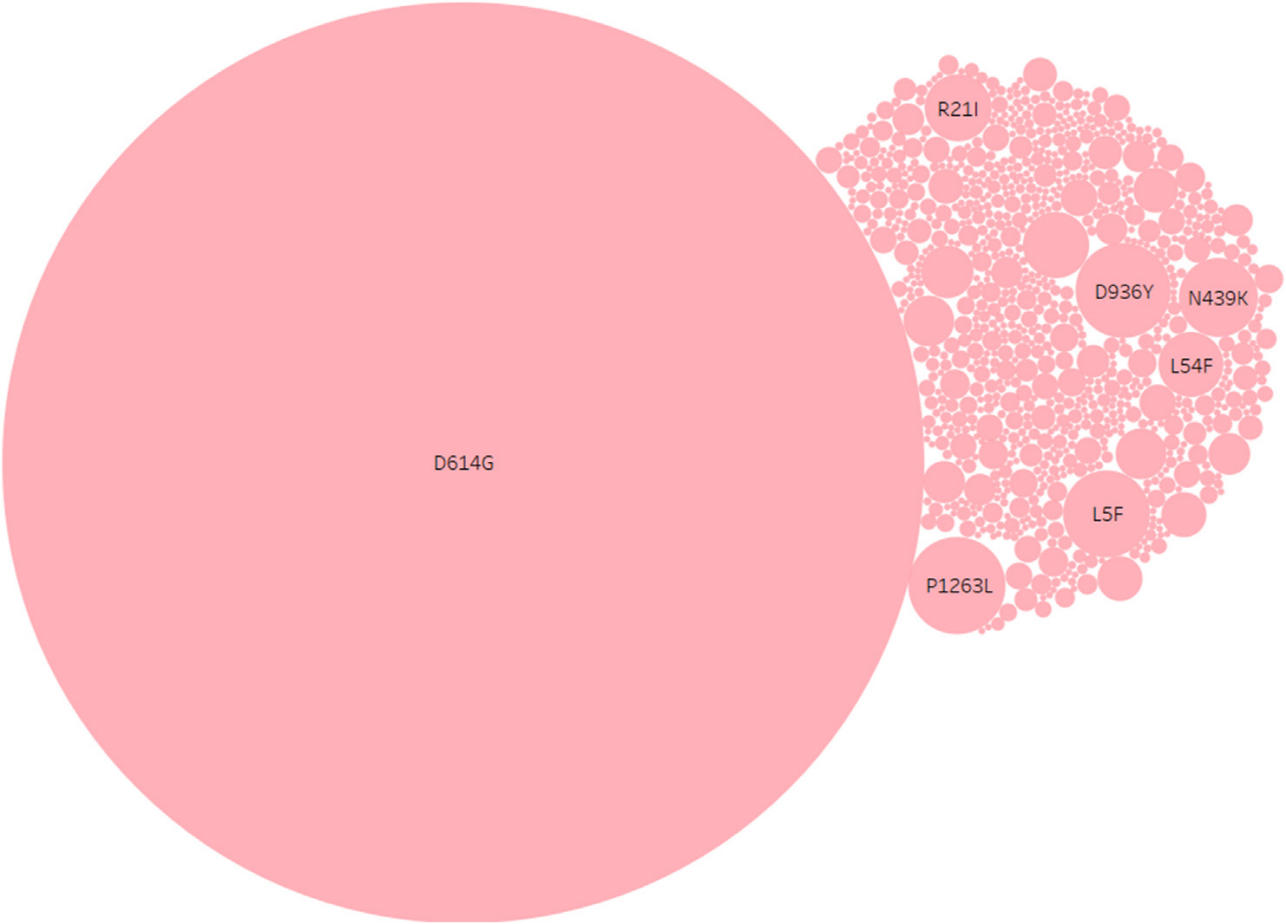
Prevalence of specific amino acid mutations among spike protein. Bubble map representing amino acid mutation and its prevalence observed among 1,44,605 genomes. Single amino acid position represents reference hCoV-19/Wuhan/WIV04/EPI_ISL_402124. Circle size shows sum or frequency of events. The marks are labelled by mutations. The mutation of Aspartic acid to Glycine at position 614 was observed 1,23,415 times among the available 1,44,605 SARS-CoV-2 spike protein sequences used in this study. Overall, the S2 domain and specially heptads repeats were rich in mutational events observed and RBM region was found least variable (0.25%) among 1273 bp long protein.

A total 1,44,604 spike glycoprotein sequences of SARS-CoV-2 genomes reported globally were retrieved from the GISAID consortium (GISAID,23/10/2020) and aligned using MAFFT with reference SARS-CoV-2 spike glycoprotein (EPI_ISL_402124) (Additional file 5) [45]. Multiple sequence alignment was studied to observe amino acid sequence variation at each residue and complete list of all amino acid variations reported (1273 aa) with number of events observed globally till were listed in additional file 5. Global analysis suggested a total 3897 mutational events reported in spike protein sequence where 1935 were observed at only a single incidence (n = 1). Among all the variations, twelve (L5F, L8V, L18F, R21I, L54F, N439K, D614G, A829T, A879S, D936Y, G1124V, P1263L) were dominant (greater than 1000 genomes) (Figure 6). Only 2 (R21I, L54F) were located at N-terminal domain (NTD), 3 variations were found in signal peptide (L5F, L8V, L18F). Single variations (N439K) were found at the receptor-binding domain (RBD) while three variations (A 829T, A879SV, and D936Y) were found at heptad repeat 1 (HR1) domain. Single variations were found in signal sub-domain-2 (D614G), sub-domain-3 and heptad repeat 2 domain (G1124V) (D1168H), and cytoplasmic tail domain (P1263L) each. Only a single variant D614G reported in 85.34% of the genome sequenced globally in 87 countries [46] (Figure 7).

#### 3.3.2 SARS-CoV-2 RBD mutation mapping

Total 29 mutations were reported in receptor binding domain of spike protein of SARS-CoV-2. Complete list of mutation, events and its origin are listed in Table 1. RBD domain showed only 0.54% of total mutational events (including n = 1) where RBM showed 0.25% frequency of mutations. The receptor-binding motif (RBM) is the main functional motif in RBD and is composed of two regions (region 1 and 2) that form the interface between the S protein and hACE2 [40].

**Table 1:** List of mutations observed in RBD domain, events and region observed among global spike protein sequences.

Sr. No	SNP	Events	Origin and initial occurrence of mutations
1	N439K	1346	Scotland, England, Romania
2	T478I	109	England
3	V483A	47	England, USA
4	G476S	24	USA, Belgium
5	S494P	41	USA, England, Spain, India, Sweden
6	V483F	29	England, Spain, Belgium
7	A475V	18	USA, Australia, England
8	F490S	9	England
9	G446S	5	England, Australia
10	E484K	15	England, Spain
11	F490L	7	Australia, USA, Singapore
12	V483I	3	England
13	Q493L	8	USA
14	G446S	5	England
15	V445I	3	England
16	E484D	3	Thailand, Germany
17	L455F	27	England
18	F456L	3	USA
19	V503F	1	USA
20	Y495N	1	Luxembourg
21	K444R	7	Spain
22	E484A	4	Spain
23	G476A	2	England
24	E484Q	21	India
25	R403K	13	Australia
26	N501Y	53	USA
27	S494L	4	Switzerland
28	Q493R	1	England
29	Y505W	18	England

#### 3.3.3 SARS-CoV-2 RBM-hACE2 interacting residue mutation mapping

Computer modelling interactions have predicted the receptor binding motif (438–506) which act as main functional motif within RBD domain contains essential seventeen key residues that are potentially involved in binding with host cell receptor ACE2 (Table 2) [39,40]. We scanned mutation at these seventeen key residues Table 2 which may impact binding of virus and host receptor [47]. Mutations at contacting residues will be important to consider for the vaccine and therapeutic targeted against S protein (RBD region) [9, 48]. Among these seventeen residues, twelve residues reported unique variations but at exceptionally low incidence (<2%) which can be due to sequencing errors or error prone replication of viruses within hosts. We observed key contacting residues were conserved amongst 99% of genomes of SARS-CoV-2 sequences.

**Table 2:** Single nucleotide polymorphism reported at key interacting residue between SARS-CoV-2 RBD - hACE2 among global spike protein sequences.

RBM Residue	ACE2 residue	RBM Residue Mutation	Number of Events
K417	Q24	K417N	1
G446	T27	G446A	1
Y449	F28	Y449N	1
Y453	D30	-	-
L455	K31	L455I	1
L456	H34	-	-
A475	E35	-	-
F486	D38	-	-
N487	Y41	N487L	1
Y489	Q42	Y489H	1
Q493	L79	Q493R	1
G496	M82	G496C	1
Q498	Y83	-	-
I500	N330	-	-
N501	K353	-	-
G502	G354	-	-
Y505	D355,R357,R393		

#### 3.3.4 Interpreting mutation effect of N439K in RBM region

To estimate the functional changes suggested by the RBM mutations, we used the prototype SARS-CoV-2 RBD domain and ACE2 and compared RBM mutants to assess their binding energy to human ACE2. The complex structure of the SARS-CoV-2 S-protein RBD domain and human ACE2 was obtained protein data bank (PDB ID: 6LZG) [49]. Mutant amino acids of the SARS-CoV-2 RBD mutants were directly replaced in the model, was used as a template modelled using SWISS-MODE [50]. We screened and evaluated interaction analysis of mutant N349K as it was most prevalent mutation observed in RBD-RBM region. RBM mutant types (N439K) exhibited significantly lowered ΔG, suggesting a slight increased affinity to human ACE2; compared to the prototype. The ΔG of these mutant types were around -11.2 kJ/mol, lower than the prototype strain (-11 kJ/mol), where 0.093 kcal.mol-1.K-1 (increase of molecule flexibility) was observed with decrease in two hydrogen bonds.

#### 3.3.5 Simulations predict that N439K variant binds tighter to human ACE2

**Figure 8:**
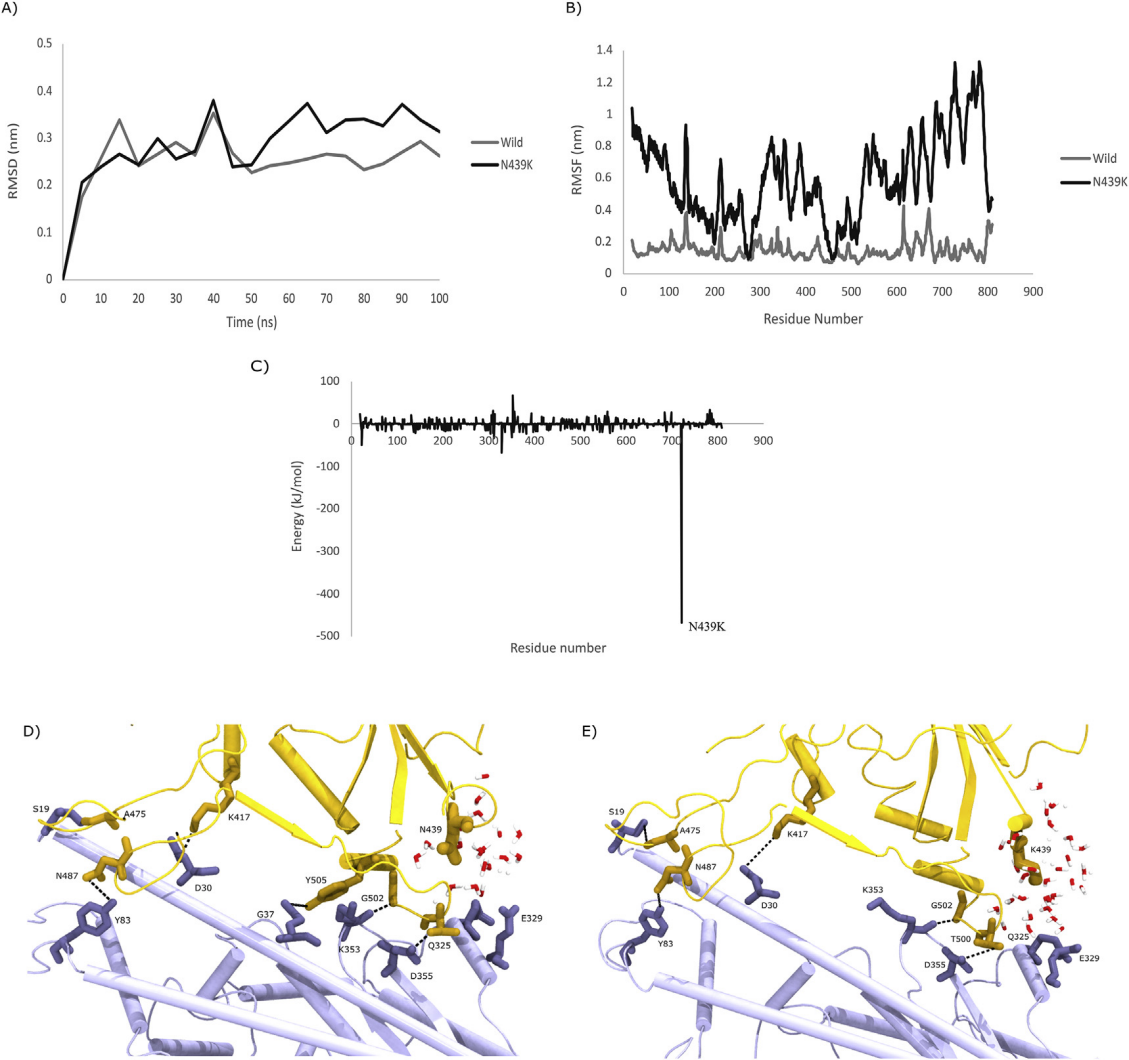
Schematic representation of RBD mutant location on RBD complexed with ACE-2 Receptor A) The RMSDs of the backbone atoms of both RBD-ACE-2 complex; B) The RMSFs of Cα atoms of both RBD-ACE2 complexes C) Binding free energies of SARS-CoV-2 RBD ACE-2 (including wild and mutant at N439K) D) Ribbon diagram structure of hydrogen bonds between SARS-CoV-2 and h-ACE2 receptor wild type E) Ribbon diagram structure of hydrogen bonds between SARS-CoV-2 and h-ACE2 receptor mutant N439K type.

As described in the Methods section, the structure of wild type Spike Receptor Binding Domain (RBD) complexed with host ACE2 receptor was retrieved from the PDB (PDB ID: 6LZG) [21]. The crystal structure of the Spike RBD complexed with ACE2 receptor reveal that the interactions are dominated by polar contacts mediated by hydrophilic residues [21]. Even a single mutation introduced in these contacts, for example, K353A could abrogate the interactions between the two proteins, highlighting the importance of the polar contacts. Thus, to understand the effect of the N439K variation, the mutant structure was built using Chimera software. Further, we performed 100 ns atomistic molecular dynamics simulations of the wild type and variant complexes. To quantify the structural variations in the protein complexes over time, the RMSD and RMSF was plotted. Both analyses indicated that the wild type of protein complex was more stable than the variant (Figure 8 A and B). On the contrary, a differential plot of the decomposed residue-wise binding free energies (Binding Free Energy of Residue _Variant -_ Binding Free Energy of Residue _Wild Type_) indicated a large change at position 439 of the Spike RBD, indicating that the residue contribution to free energy at this position was much more favorable in the variant than the wild type (Figure 8 C). A closer analysis indicates that in the presence of Lysine at position 439 of Spike protein forms water mediated hydrogen bonds with residues Gln 325 and Glu 329 of the ACE2 protein which are more distant in the presence of Asn at position 439 (Figure 8 D and E). Thus, simulations coupled with binding free energy calculations suggest that the N439K variant binds ACE2 tighter than the wildtype.

### 3.4 Interpreting N439K in RBM region in the context of antibody epitopes

The RBD is the dominant target of neutralizing antibodies to SARS-CoV-1 also, SARS-CoV-2 [51,52]. RBD-targeting antibodies could neutralize binding SARS-CoV-2 with ACE2 [57]. Several antibodies that target the RBD of SARS-CoV-2 have now been reported in very recent studies [53, 54, 55]. It is unclear to what extent the RBM will evolve to escape neutralizing antibodies in a manner evocative of vaccine escape mutants [58]. Many antibodies have epitopes that overlap the RBD ACE2 contact interface and are therefore strongly constrained by mutation effects RBM region [Figure 8], such as B38, BD23, CB6,P2B–F26, EY6A and REGN 10933 [[12], [13], [14], [15],^51^,^54^,^56^]. To better define the RBM's mutations N439K for antibody escape, we examined structural and binding constraint in the epitopes of antibodies with prototype structures of SARS-CoV-2 RBD.

Structural binding predictions suggests that prevalent mutation in spike RBD region, N439K did not overlap with currently characterized epitopes of neutralizing antibodies recovered from human convalescent patient [47, 48, 49]. The importance of tracking such mutations within RBM region is demonstrated by a recent study that identified mutations enabling escape from RBD-directed neutralizing antibodies [60]. Our data indicate that the none of the currently characterized mutations within RBM region escape the five RBM targeted neutralizing antibodies used in this study.

## 4 Discussion

COVID-19 pandemic since its emergence in China has been showing varied patterns of transmission and infectivity globally [61, 62]. Studies on global diversity and real time tracking of mutations will be important in the disease control strategies [59, 63, 64, 65]. India represents an excellent opportunity for such studies for its size, observing a strictest complete lockdown and varied environmental conditions. Many studies have reported the genomic characterization of Indian isolates till date with limited sample size [66]. We present here a complete comprehensive genomic characterization of pan India SARS-CoV-2 genomes till 23/10/2020 with more than eight months of pandemic, with an objective of understanding its global positioning. The study suggests similarity of Indian genomes with predominant types found across different parts of the globe [67]. It was noted that D614G mutant strains in India increased rapidly during eight months of the pandemic and is present in more than 81% of the genome sequences reported from India. The recent studies by Korber et al [27] and WHO collaborating study in China demonstrated D614G strains are 10-fold more infectious than the original Wuhan-1 strain [69]. There are reports suggesting associations of increased prevalence of G614 variant to the higher case fatality rates in 12 different countries [70]. In India, 81.66 % of genomes exhibited G614; however, its association with increased CFR could not be discovered in India. Distinctively, Indian isolates also did not displayed emergence of co-evolving mutations associated with mutant strain G25563T (ORF3a), C26735T (NSP14) and C18877T (M protein), which are observed globally. The study also attempted to look for such correlations in Indian states which exhibited highest mortality. Yet even in such states, the association with D614G could not be found.

Recently, the emergent UK variant strain named as VUI-202012/01 with defined as a set of 17 mutations, more significant N501Y was also observed and reported in India. As per recent studies, the UK variant reported higher binding affinity towards RBD region of spike protein. Preliminary modelling results communicated by the UK on 19^th^ December suggested that the variant is significantly more transmissible than previously circulating variants, with an estimated increase in reproductive number (R) by 0.4 or greater with an estimated increased transmissibility of up to 70% [78].The variant has proven to transmit more easily and had spread to 10 more countries over the short time. Similarly, another variant GH/501Y.V2,B. 1.351, famously known as South African Variant have been reported. The variant has spread to 31 countries and is shown to be responsible for higher fatality rates. Recently, four cases with the South Africa variant of SARS-CoV-2 were reported in India. Also, one case of Brazil variant known as 20J/501Y.V3 or P.1 lineage was also reported recently. India is reporting a spurt in the number of cases in some states. However, it needs to be ascertained whether this increase is related to emergence of UK, South African, Brazilian variants, or new Indian variants. To this effect, Indian Govt have launched Indian SARS-CoV-2 Genomic Consortia (INSACOG) which will accelerate virus surveillance, genome sequencing and characterization through a multi-laboratory network.

Many therapeutics are currently being pursued with the goal of developing protective immunity against the SARS CoV-2 virus [55, 72]. Notably, immunogens and diagnostics are under development targeting spike protein sequence from the Wuhan reference strain. Several spike protein mutant strains are being reported so far. This study analysed 1, 44,605 spike protein sequences reported globally. Despite of a total of 3895 mutational events observed in spike protein sequences, frequency of events and its occurrence is still low in total available sequences except for D641G. RBD being an important region in host-virus interaction and thus for development of therapeutics, frequency of mutational events observed was very low [68]. The prevalence of mutations in RBD was found low (~0.57%) as compared to genome sequences reported globally. Single variant N439K at RBD region was observed in 0.52% (n = 293) of genomes. Notably, N439K mutant is reported to show increased affinity binding affinity with hACE2. Interestingly, Indian isolates did not show presence of this variant. It is further noted that this variant is reported majorly few countries, including Scotland, England, and Romania. This led us to study the impact of observed N439K mutation within RBM region using computational docking and MD simulations approaches. Using a panel of RBM directed monoclonal antibodies viz. B38, BD23, CB6, P2B–F2F, and EY6A [12, 13, 14, 15, 51, 54, 56], study predicts slightly higher affinity to ACE receptor. This needs further confirmatory studies using *in-vivo* and *in-vitro* studies.

One of the reasons for lack of association of genomic signatures of Indian isolates with fatality rates could be low sample size. Large scale monitoring of Indian isolates is required for conclusive correlations. The data from this study along with serosurveillance data from India and clinical meta data will be useful to decipher associations between viral clades and severity. The approach and global positioning of Indian isolates presented in this study will be helpful for monitoring the pandemic in India. Several other factors for low susceptibility of Indians to COVID-19 are being also being proposed. Such factors include host innate immunity status, genetic diversity in immune responses, epigenetic, ABO blood group association and universal BCG immunization need to be investigated with the clinical studies.

## 5 Conclusions

The study suggests that SARS-CoV-2 virus diversity in India is consistent with global trends with respect to most prevalent mutations. No major mutation event was reported in RBD region of studied Indian isolates. Global spike protein mutation prevalence analysis suggests impact of some mutations on RBD and ACE receptor interaction which may affect virus infectivity. The study findings are cautiously optimistic that viral diversity will not be hindrance to current vaccine development strategies and will be broadly effective against current isolates.

## Declarations

### Author contribution statement

Sunil Gairola: Conceived and designed the experiments; Analyzed and interpreted the data; Contributed reagents, materials, analysis tools or data; Wrote the paper.

Shweta Alai: Conceived and designed the experiments; Performed the experiments; Analyzed and interpreted the data; Wrote the paper.

Nidhi Gujar: Performed the experiments.

Manali Joshi: Analyzed and interpreted the data; Contributed reagents, materials, analysis tools or data; Wrote the paper.

Manish Gautam: Conceived and designed the experiments; Analyzed and interpreted the data; Contributed reagents, materials, analysis tools or data; Wrote the paper.

### Funding statement

This research did not receive any specific grant from funding agencies in the public, commercial, or not-for-profit sectors.

### Data availability statement

Data associated with this study has been deposited at GISAID under the accession number provided in additional information.

### Declaration of interests statement

The authors declare no conflict of interest.

### Additional information

No additional information is available for this paper.

## Appendix A Supplementary data

Additional File 1 The complete characteristics of 2525 SARS-CoV-2 genomes sequences used in the study. Additional File 1

Additional File 2 The state wise list of mutations observed in country. Additional File 2

Additional File 3 The clade and lineage of SARS-CoV2 genomes from India. Additional File 3

Additional File 4 Lineages observed India are clustering with sequences from Asia, Europe, the USA, and other Asian countries indicating multiple introductions of multiple lineages of the virus into the country. Additional File 4

Additional File 5 A total 1,44,605 Spike glycoprotein sequences of SARS-CoV-2 genomes reported globally were retrieved from the GISAID consortium. Additional File 5
